# Transdiagnostic internet cbt for mixed anxiety and depressive: Results from a feasibility study in primary care

**DOI:** 10.1192/j.eurpsy.2021.930

**Published:** 2021-08-13

**Authors:** P. Roberge, H.-M. Vasiliadis, A. Lesage, R. Labelle, H. Haskelberg, J. Grenier, M. Drapeau, M.D. Provencher

**Affiliations:** 1 Department Of Family And Emergency Medicine, Université de Sherbrooke, Sherbrooke, Canada; 2 Department Of Community Health Sciences, Université de Sherbrooke, Longueuil, Canada; 3 Psychiatry, Université de Montréal, Montreal, Canada; 4 Psychology, Université du Québec à Montréal, Montreal, Canada; 5 Clinical Research Unit For Anxiety And Depression, St. Vincent’s Hospital, Syndney, Australia; 6 Institut Du Savoir Montfort, Hôpital Montfort, Ottawa, Canada; 7 Educational And Counselling Psychology, McGill University, Montreal, Canada; 8 School Of Psychology, Université Laval, Québec, Canada

**Keywords:** Digital therapy, anxiety and depressive disorders, primary care, Cognitive-Behaviour Therapy

## Abstract

**Introduction:**

In response to the treatment gap for anxiety and depressive disorders, psychological treatments with innovative modalities and high implementation potential are essential. Internet CBT (iCBT) is a cost/effective approach that could improve access to a low-intensity evidence-based CBT intervention.

**Objectives:**

To assess the feasibility and acceptability of the French adaptation of the physician-prescribed six-lesson This Way Up transdiagnostic iCBT program for mixed anxiety and depressive disorders developed in Australia.

**Methods:**

Feasibility study with pre- post-intervention evaluations, including an embedded qualitative study in Family Medicine Groups (Quebec, Canada). Inclusion criteria comprise a family physician diagnosis of Major Depression, Panic Disorder, Agoraphobia, Social Anxiety Disorder or Generalized Anxiety Disorder. Primary self-reported outcomes: PHQ-9 (depression) and GAD-7 (anxiety); secondary measures include diagnostic-specific scales and health service utilisation.

**Results:**

Family physicians (N=21) from five Family Medicine Groups prescribed iCBT to 45 patients (30 women, 15 men; mean age = 39.7), 31 initiated the program. To date, 20 patients completed 5 or 6 lessons, nine completed between 2 and 4. Intervention and post-treatment assessments are ongoing, results forthcoming. Results of semi-structured interviews with patients (N=15) and family physicians (ongoing) on iCBT acceptability indicate it is beneficial, practical and easy to use. Program adherence requires patient readiness and determination and could be fostered by motivational support from clinicians.

**Conclusions:**

Results support this French iCBT program’s scaling-up potential to contribute to reducing the gap in evidence-based treatments for common mental disorders. Its implementation in primary care could improve the effectiveness, efficiency and equity to a rapidly accessible treatment.

